# The Institutional Treatment of Consumption

**Published:** 1916-07-15

**Authors:** 


### THE INSTITUTIONAL TREATMENT OF CONSUMPTION.

### VI.—Records and Classification of Patients.*

A record of the physical condition of a patient on admission is obviously an important document, and every endeavour is made in sanatoria to compile such records accurately and to embody in them an informative history of the patient's previous health and subsequent progress. The authorities of most sanatoria, in addition to publishing statistics and analyses of their " results " of treatment, endeavour to keep in touch for a number of years with their old patients. After-histories are valuable and have been of assistance in estimating the worth of systematic treatment. Needless to say the task of following up patients is a difficult and often thankless one, but there is no other way of obtaining what is, after all, useful information.

History sheets usually are more or less stereotyped in plan, in order to conduce to uniformity and to remind easily the medical officer of the set questions. In this way a brief summary is made of the patients' previous health, the mode of onset of the disease, duration, possible source of infection, and previous treatment. The family history is also epitomised, and a note is made of the environment and usual occupation of the patient. The present condition is naturally carefully analysed, both as to symptoms and physical signs. Of the former the following are the chief ones inquired into: cough, expectoration, pain, hæmoptysis, wasting, dyspnœa, diarrhœa, and dyspepsia. The physical signs in the lungs, the outward conformation of the chest, etc., are recorded on a chart which gives an outline of the ribs, on which may or may not be superimposed a diagram of the lungs and their lobar subdivisions. Many diagrams in use are too cramped to allow for accurate markings, or else are overloaded with outlines too thick to allow the physician's signs to show up clearly. An outline of the glottis is usually also printed for recording easily the result of laryngoscopy.

### Wanted, a Standard List of Signs.

It is a very great pity that a standard list of signs has not been agreed upon and universally adopted. The great impetus given to the treatment of consumption should have been taken advantage of as an opportunity for the adoption of a simple list of diagrammatic signs and abbreviations. Some attempt has been made in this direction, but anyone who has to study the records made by general practitioners and tuberculosis officers on the admission sheets of, for example, insured persons, will agree that there is a lack of uniformity and a consequent ambiguity which greatly lessens the value of these records. Beyond the recognition of dots as denoting râles, the conventional signs are not used in such a way as to convey definite phenomena or to be interpreted in the same way by every medical man. There is a widely-recognised international code of signs which could easily be adopted; the most frequently used abbreviations are quickly learned, and are not burdensome to carry in the mind. Now that so much form-filling has to be done by medical practitioners and tuberculosis officials, it would be a positive gain if some degree of uniformity in this respect could be obtained. There is also need for a uniform record sheet on which the history of the patient's illness, the classification, etc., would be recorded in the same way in every district, so that with the transference of a patient from one district to another, his tuberculosis records could be readily assimilated in the files of the new locality.

In sanatorium records more attention should be devoted to the progress of the patient towards restoration of general health and capability of performing work than to repeated statements detailing the extent and activity of the physical signs in the lungs. After the first careful examination there should be no need to insist, as is done in some institutions, on a routine examination of every patient's chest at fortnightly intervals. Special examinations in connection with the onset of fresh symptoms or just before discharging a patient will always be required, but better information concerning progress can be obtained by watching the effect of the tuberculo-toxin on the patient's general condition as shown by the temperature chart, the quantity of sputum, the weight, the appetite and general well-being of the subject. An examination of the chest about once a month in an ordinary case should be sufficient.

### Examinations for Tubercle Bacilli.

If tubercle bacilli have been found in the sputum the diagnosis is clinched, and until the patient is nearing the end of his stay the sputum need not be examined afresh. A search for T.B. must then again be undertaken in order to ascertain if the patient is " infectious " or no. The presence of T.B. must be read in conjunction with the state of the lesions in the lungs, for bacilli in the sputum do not necessarily imply activity; patients may be " carriers " analogous to the typhoid carriers, and are described on discharge as " Arrested disease + T.B.," that is to say, their health is restored but they are capable of infecting others. On the other hand, if on admission no T.B. are found in the sputum, repeated examination of the expectoration is called for, and one of the concentration methods is usually employed. All negative examinations must be accepted with reserve if there are other suspicious factors in the case, and seven or eight examinations of the sputum may be made before bacilli are found. Even absence of sputum is not evidence of freedom from disease, for not every tuberculous focus breaks down.

To return to the records. An indispensable item is a method of classifying patients, so that by means of a symbol any person's state on admission shall be denoted with sufficient clearness to convey

* The previous articles appeared on May 6 and 20, June 3 and 17, and July 1, 1916.

a definite impression to his medical attendant. From what has been said it will be gathered that such a set of symbols is not an easy matter to arrange, and hence several classifications are in vogue. By denoting only the extent of the lesions in the lungs, the far more important factor of the patient's health and resistance is omitted. A patient with extensive cavities may be capable of a hard day's work, while a patient with a very small lesion may be extremely ill. The three best-known classifications are those known as Turban's, Philip's, and the Departmental Committee's classifications.

Turban's method is anatomical, and gives three stages or classes. *Stage I.* denotes disease of slight severity affecting at most one lobe or two half-lobes. *Stage II.* denotes disease of slight severity affecting at most two lobes or severe and affecting at most one lobe. *Stage III.* includes all cases of greater extent and severity than those in the second stadium.

The Departmental Committee's classification, which appeared in the interim report, deals with the working capacity of patients and their requirements as regards hospital and sanatorium accommodation. It is of use for administrative purposes. There are six classes as follows:—

1. Cases in which the disease can be diagnosed or strongly suspected, but in which there is no evident impairment of the working capacity.
2. Cases of recent onset, with some impairment of the working capacity, but without marked evidence of ill-health.
3. Cases of recent onset, with evidence of acute illness.
4. Cases of a longer history of illness. In some of these cases permanent arrest of the disease may be hoped for, but in the majority restoration to full working capacity for more than a comparatively short time is not to be hoped for.
5. Cases in which there is permanent loss of working capacity. Many of these patients live for a considerable time in a condition of chronic ill-health.
6. Cases in which a fatal termination within six months is probable.

PHILIP'S CLASSIFICATION.

The clinical method is better represented by Philip's classification, which gives due expression to the local mischief, which is denoted by the symbol L, and to the degree of systemic intoxication denoted by the symbol S. Sir Robert Philip in his own classification specified no less than twelve divisions effected by using small letters (l, s,) as well as capitals. A commonly used modification allows for only six classes, and this can be made to adapt itself to the great majority of cases. These six are $L_1s$; $L_1S$; $L_2s$; $L_2S$; $L_3s$; $L_3S$. The local lesion is practically classified after Turban's method by the addition of the numerals, and the amount of systemic intoxication is denoted *comparatively* by the large S or the small s. Those cases which show no obvious symptoms of ill-health may be denoted by $L_1$; $L_2$; $L_3$; without an s, thus giving three more classes. And again some tuberculosis officers find it advantageous to denote by the symbols $l_1S$; $l_2S$; $l_3S$ those cases where the systemic infection is much greater proportionally to the discovered lesions. It will be soon gathered in practice that the six classes first referred to above will serve most occasions, that the three L's without an s are useful, and that the last three can be dispensed with without much disadvantage.

Which of these three classifications will be most generally adopted and eventually receive official sanction remains to be seen, but it is to be hoped that the Turban method will not be the one. Attempts have been made to correlate the Departmental Committee's classification with that of Philip's, but such correspondence as has been suggested is unsatisfactory in practice and must vary according to the personal views of the observer. For instance, Class I. may be correlated with either $L_1$ or $L_1s$, and Class II. with $L_1s$ or $L_2s$. It seems probable that Philip's method will increase in popularity.

Where a system of graduated exercise and labour is carried out a classification is used which is governed by the factor of auto-inoculation. For example: *Class I.* No auto-inoculation even after hard work. *Class II.* Able to go through the grades with only occasional and controllable inoculations. *Class III.* Able to be out of bed, but in whom even walking exercise is accompanied by fever and constitutional symptons. *Class IV.* Those patients who quickly recover from the effects of auto-inoculation when completely immobilised. *Class V.* Those who are continually suffering from inoculation even when on complete rest.

